# Influence of Uncomplicated Phacoemulsification on Central Macular Thickness in Diabetic Patients: A Meta-Analysis

**DOI:** 10.1371/journal.pone.0126343

**Published:** 2015-05-12

**Authors:** Jianping Liu, Richard Edward Jones, Jiangyue Zhao, Jinsong Zhang, Fan Zhang

**Affiliations:** 1 Department of Ophthalmology, The Fourth Affiliated Hospital of China Medical University, Shenyang, Liaoning, People’s Republic of China; 2 Department of Ophthalmology and Vision Science, University of Arizona College of Medicine, Tucson, Arizona, United States of America; 3 Eye Hospital of China Medical University, Shenyang, Liaoning, People’s Republic of China; 4 Key Lens Research Laboratory of Liaoning Province, Shenyang, Liaoning, People’s Republic of China; University of Utah (Salt Lake City), UNITED STATES

## Abstract

**Objective:**

To evaluate the effect of uncomplicated phacoemulsification on central macular thickness (CMT) and best corrected visual acuity (BCVA) in both diabetic patients without diabetic retinopathy (DR) and diabetic patients with mild to moderate non-proliferative diabetic retinopathy (NPDR).

**Methods:**

Potential prospective observational studies were searched through PubMed and EMBASE. Standardized mean difference (SMD) and 95% confidence interval (CI) for changes in CMT and BCVA were evaluated at postoperative 1, 3 and 6 months. The pooled effect estimates were calculated in the use of a random-effects model.

**Results:**

A total of 10 studies involving 190 eyes of diabetic patients without diabetic retinopathy and 143 eyes of diabetic patients with NPDR were identified. CMT values demonstrated a statistically significant increase after uncomplicated phacoemulsification at 1 month (SMD, -0.814; 95%CI, -1.230 to -0.399), 3 months (SMD, -0.565; 95%CI, -0.927 to -0.202) and 6 months (SMD, -0.458; 95%CI, -0.739 to -0.177) in diabetic patients with NPDR. There was no statistical difference in CMT values at postoperative 1 month (SMD, -1.206; 95%CI, -2.433 to 0.021)and no statistically significant increase in CMT values at postoperative3 months (SMD, -0.535; 95%CI, -1.252 to 0.182) and 6 months (SMD, -1.181; 95%CI, -2.625 to 0.263) in diabetic patients without DR.BCVA was significantly increased at postoperative 1 month (SMD, 1.149; 95%CI, 0.251 to 2.047; and SMD,1.349; 95%CI, 0.264 to 2.434, respectively) and 6 months (SMD, 1.295; 95%CI, 0.494 to 2.096; and SMD, 2.146; 95%CI, 0.172 to 4.120, respectively) in both diabetic patients without DR and diabetic patients with NPDR. Sensitivity analysis showed that the results were relatively stable and reliable.

**Conclusion:**

Uncomplicated phacoemulsification in diabetic patients with mild to moderate NPDR seemed to influence significantly the subclinical thickening of the macular zones at postoperative 1, 3 and 6 months compared with diabetic patients without DR. BCVA was significantly improved in both diabetic patients without DR and diabetic patients with mild to moderate NPDR.

## Introduction

Cataracts are the most common cause of blindness in the world, and they usually require surgical removal [1]. The worldwide prevalence of diabetes is on the rise, and patients with diabetes have higher risk of developing cataract compared with patients without diabetes [2]. At present, the main surgical procedures are phacoemulsification and posterior chamber intraocular lens implantation [3]. Cystoid macular edema (CME) is one of the main causes of unfavorable visual outcomes and one of the most common complications following uncomplicated cataract surgery in patients with and without diabetes, which is measured by an alteration in central macular thickness (CMT) using optical coherence tomography (OCT) [4]. Several reasons may be proposed as underlying pathogenicmechanisms of macular thickening; for instance postoperative inflammation caused by surgically damaged tissue, breakdown of the blood—retinal and blood-aqueous barriers, or the release of prostaglandins and vascular endothelial growth factor (VEGF) [4,5]. Cataract surgery is an inflammatory insult to the eye, and the risk of macular thickening after uncomplicated phacoemulsification may increase in the presence of ocular or systemic diseases such as uveitis or diabetes [6]. The incidence of pseudophakic CME has been reported in healthy populations and in patients with diabetes after uncomplicated phacoemulsification [7]. Progression of clinically significant macular edema with visual impairment frequently can be observed in diabetic patients, especially in those with preexisting proliferative retinopathy after uncomplicated phacoemulsification [8]. Diabetic retinopathy (DR) is a common microvascular complication of diabetes, resulting in increased permeability of retinal blood vessels and swelling of the macula [9]. Some studies have shown that the severity of retinopathy may have an influence on the visual outcomes after uncomplicated phacoemulsification in patients with diabetes [10,11]. Unstable DR with clinically significant macular edema at the time of phacoemulsification surgery tends to worsen postoperative macular edema [12]. Currently, there is little robust evidence to show the effect of uncomplicated phacoemulsification on the changes of CMT in patients with different levels of severity of retinopathy.

The aim of this study is to assess the impact of uncomplicated phacoemulsification on the changes of CMT values and BCVA in both diabetic patients without DR and diabetic patients with mild to moderate non-proliferative diabetic retinopathy (NPDR).

## Materials and Methods

### Search strategy

Relevant literature was obtained through PubMed and EMBASE databases (most recently updated in August, 2014) for prospective observational studies reporting related values of CMT and BCVA in diabetic patients without DR or in diabetic patients with NPDR after uncomplicated phacoemuification using the search terms “macular thickness”, “cataract surgery” and “diabetes”. The literature was searched without language limitation. Relevant references were retrieved if they met the objective of this meta-analysis. This study was carried out with approval from the Institutional Review Board of The Fourth Affiliated Hospital of China Medical University and complied with the tenets of the Declaration of Helsinki.

### Study selection

We identified potential studies if they met the following criteria: (1) prospective observational studies; (2) Patients with type 2 diabetes mellitus who were diagnosed with cataract and underwent uncomplicated phacoemulsificationand posterior chamber intraocular lens implantation; (3) diabetic patients without diabetic retinopathy or diabetic patients with mild to moderate NPDR; and (4) basic data to calculate these values (e.g., CMT, BCVA). Exclusion criteria were the presence of additional underlying diseases other than diabetes and cataract that could affect macular thickness (e.g., uveitis, glaucoma, or epiretinal membrane); proliferative diabetic retinopathy or preexisting macular edema; no baseline data, no aggregate results, double reported, and unrelated outcome measurements.

### Data collection and quality assessment

The following information was extracted from the selected studies: each study’s first author, publication year, study design, study location, mean age of patients, measurement of outcome for CMT or BCVA, sample size at final follow-up, severity of diabetic retinopathy, quality control, and follow-up periods. If there was a disagreement in which studies should be included in this study, consensus was made by discussion among the research group. We evaluated the methodological quality of eligible studies using the Newcastle-Ottowa Scale for observational studies [13].

### Data analysis and synthesis

Data synthesis and analysis was conducted as described in detail previously [14]. The statistical analysis was performed using Stata 10 (Stata Corp LP, College station, TX). Results were presented as standardized mean difference (SMD) with 95% confidence interval (CI) using random-effects models. Heterogeneity among studies was analyzed by the Chi-squared statistic [15]. To explore the stability and reliability of our results, we evaluated the influence of each individual study on the pooled effect size by a sensitivity analysis. Potential publication bias was assessed with the Egger's regression asymmetry test [15].

## Results

### Literature search

**Fig 1:**
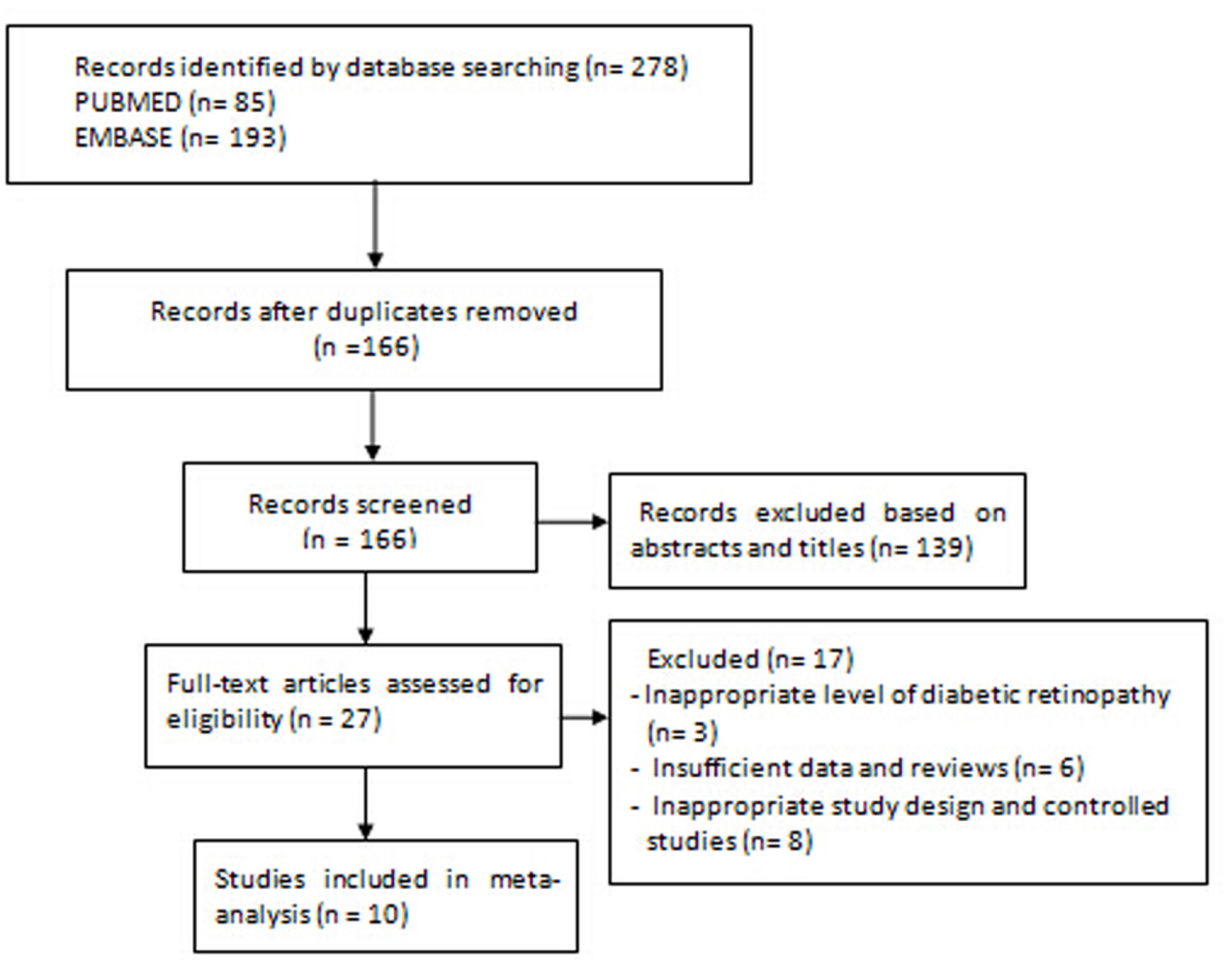
Flow chart of study selection process.

A total of 166potentially relevant articles were retrieved after removing duplicates. 139 articles were excluded after first-pass review of titles and abstracts. 17 studies were further excluded after full text review according to the inclusion and exclusion criteria specified above. The flow chart of literature search strategy is shown in Fig 1. Thus, 10 prospective studies [8,11,16–23] were identified. The characteristics of eligible studies are described in Table 1.

**Table 1:** Characteristics of enrolled studies in the meta-analysis.

Author	Year	Country	Study design	No. eyes NDR/NPDR	Mean age (years)	Time of follow-up (months)	Quality control	Level of retinopathy
Katsimpris JM et al.	2012	Greece	Prospective observational study	49/NR	68.3	1, 3, 6	8	NDR
Garcia-Martin E et al.	2013	Spain	Prospective observational study	35/NR	69.8	1	7	NDR
Hayashi K et al.	2009	Japan	Prospective observational study	34/34	67.9	3, 6	9	NDR/mild to moderate NPDR
Hartnett ME et al.	2009	USA	Prospective observational study	19/6	67.5	1, 6	8	NDR/mild to moderate NPDR
Giocanti-Aurégan A et al.	2013	France	Prospective observational study	21/NR	70.6	3, 6	7	NDR
Tsilimbaris M et al.	2012	Greece	Prospective observational study	NR/27	65.95	1, 3, 6	8	mild to moderate NPDR
Eriksson U et al.	2011	Sweden	Prospective observational study	NR/34	71	6	7	mild to moderate NPDR
Biró Z et al.	2010	Hungary	Prospective observational study	NR/18	64.3	1	7	mild to moderate NPDR
Degenring RF et al.	2007	Germany	Prospective observational study	NR/24	72.7	1	7	mild to moderate NPDR
Pierru A et al.	2014	France	Prospective observational study	32/NR	76	1, 3	8	NDR

No., number; NDR, no diabetic retinopathy; NPDR, non-proliferative diabetic retinopathy; NR, not reported.

### Meta-analysis

**Fig 2:**
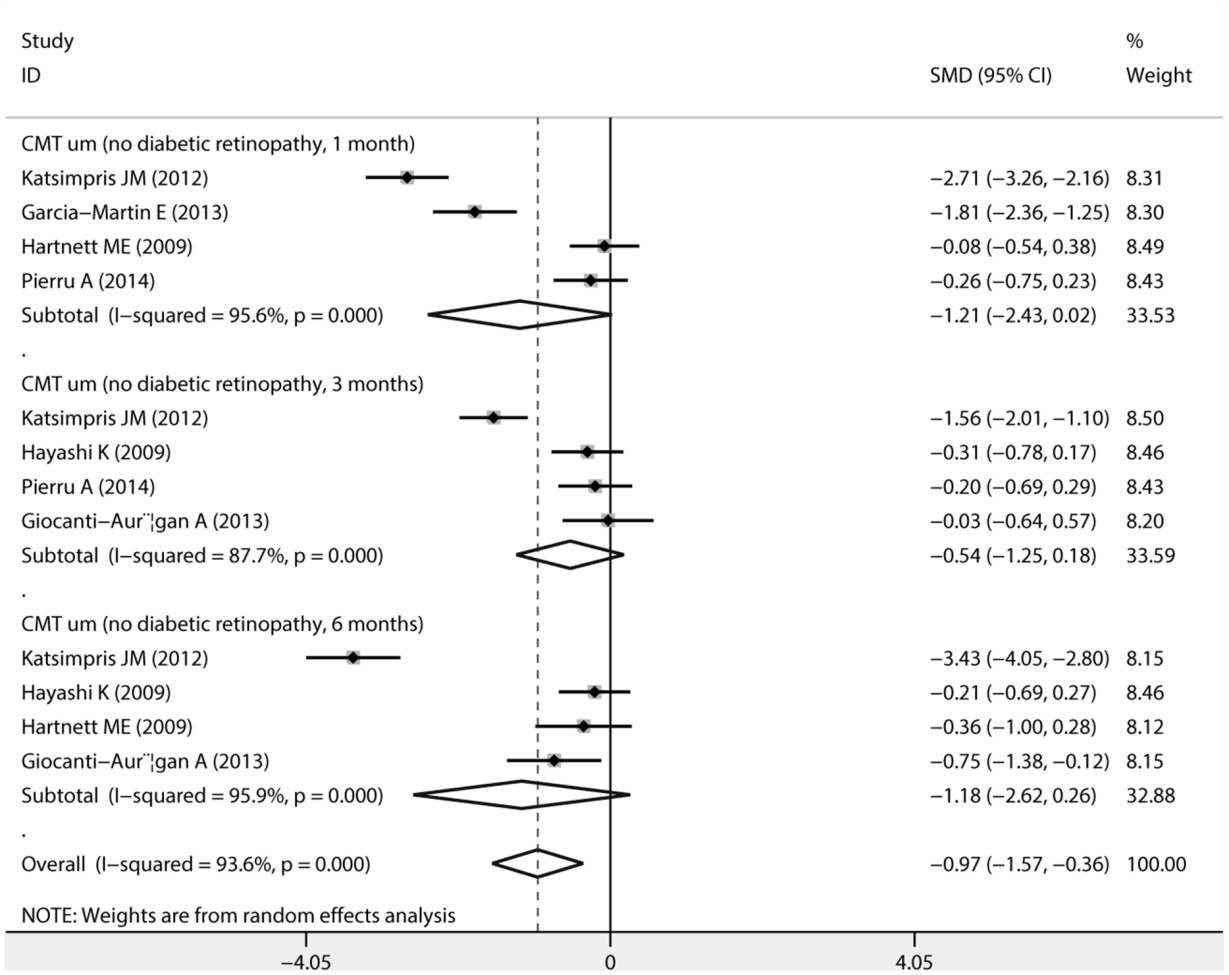
Forest plots of random-effects for pooled SMDs of central macular thickness (CMT) after uncomplicated phacoemulsification in diabetic patients without diabetic retinopathy.

The pooled estimates of mean changes in CMT (um) values after uncomplicated phacoemulsificationin diabetic patients without DR along with SMD and 95% CI are showed in Fig 2. There was no statistical difference in CMT values at postoperative 1 month (SMD,-1.206; 95%CI, -2.433 to 0.021; P = 0.054), 3 months (SMD, -0.535, 95%CI, -1.252 to 0.182, P = 0.143),or 6 months (SMD,-1.181; 95%CI, -2.625 to 0.263; P = 0.109).

**Fig 3:**
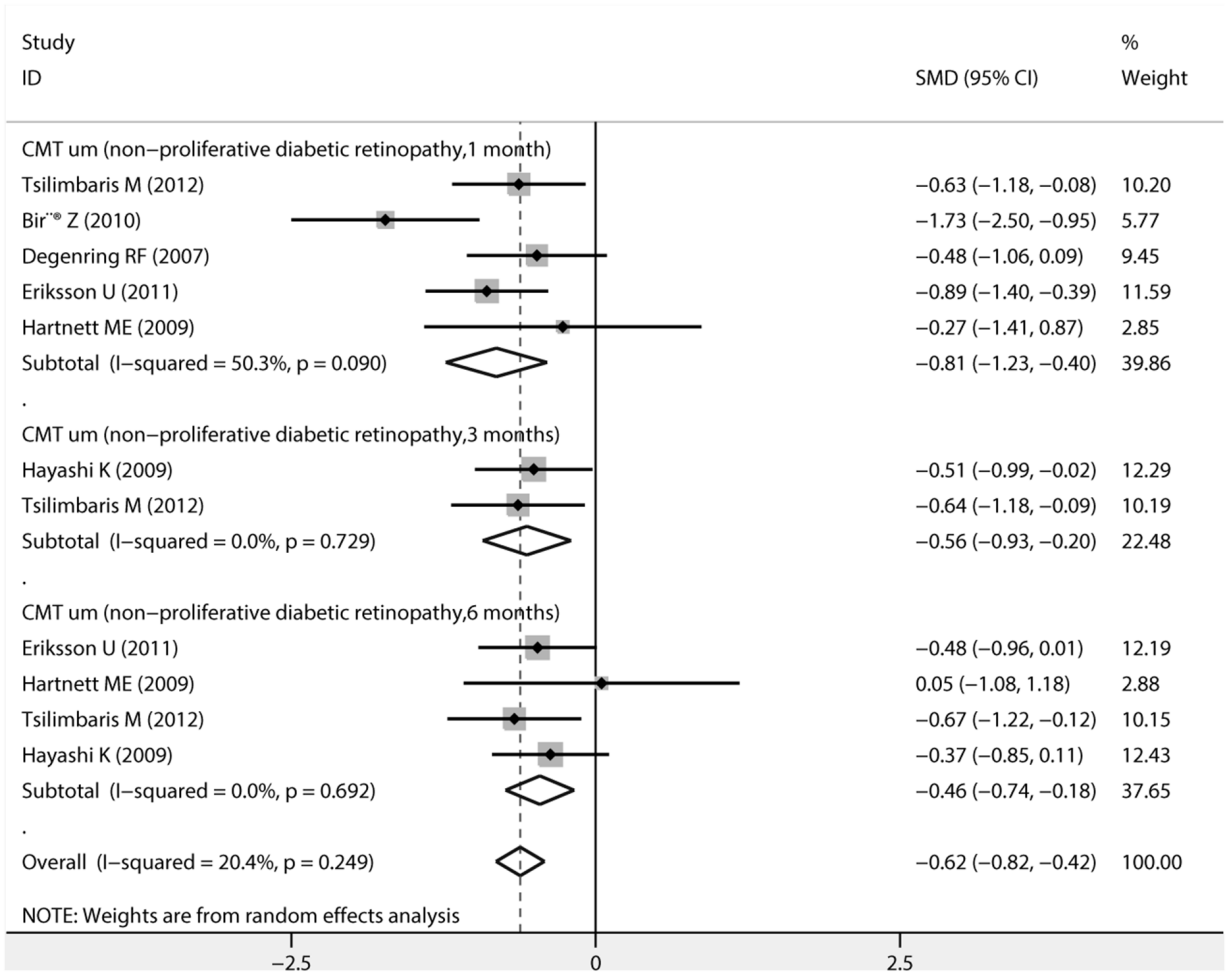
Forest plots of random-effects for pooled SMDs of central macular thickness (CMT) after uncomplicated phacoemulsification in diabetic patients with mild to moderate non-proliferative diabetic retinopathy.

The pooled estimates of mean changes in CMT (um) values after uncomplicated phacoemulsification in diabetic patients with mild to moderate NPDR, along with SMD and 95%CI are shown in Fig 3. A significant increase in CMT values were found at postoperative 1 month (SMD, -0.814; 95%CI, -1.230 to -0.399, P<0.001),3 months (SMD,-0.565; 95%CI, -0.927 to -0.202, P = 0.002) and 6 months (SMD, -0.458; 95%CI, -0.739 to -0.177, P = 0.001).

**Fig 4:**
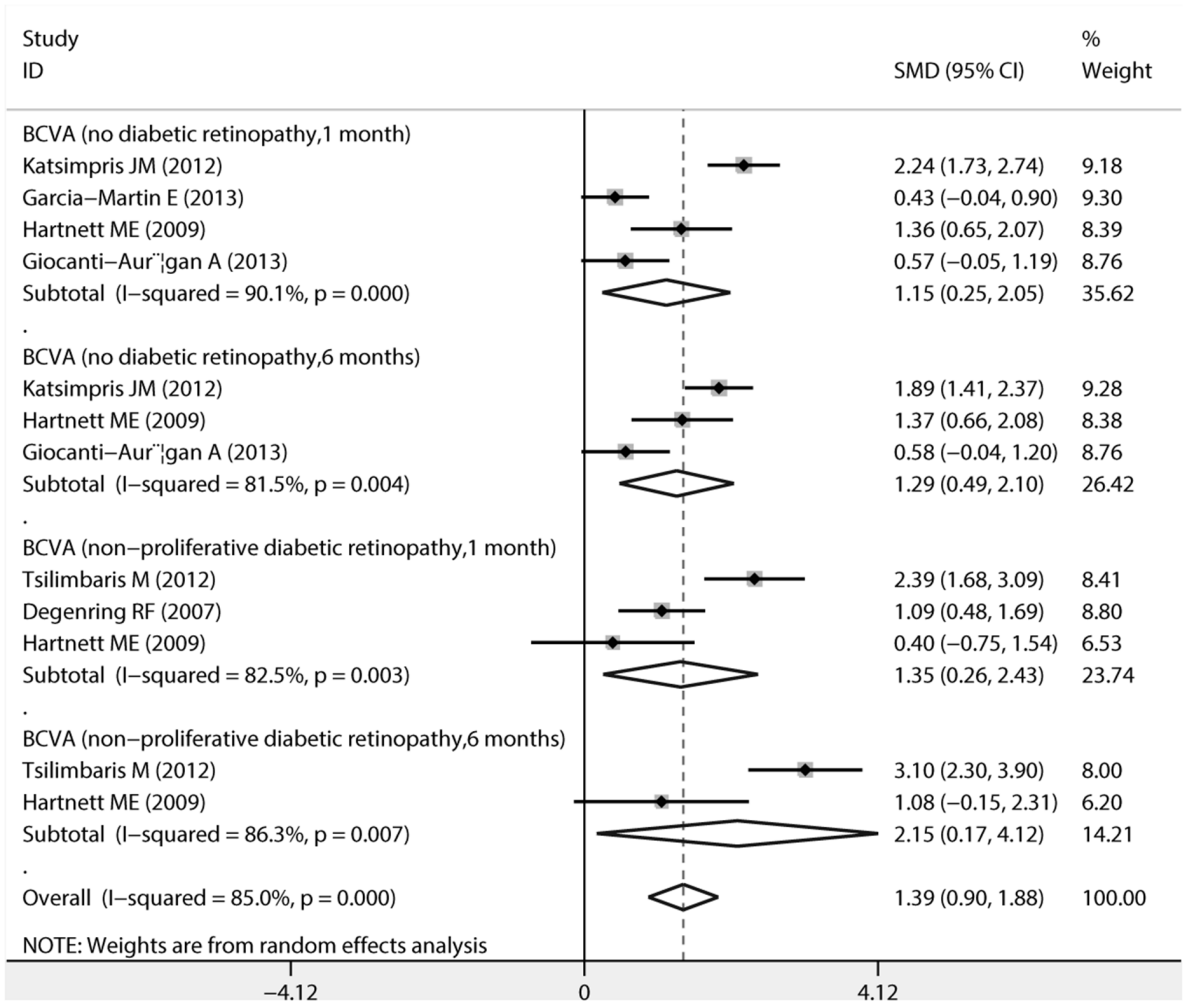
Forest plots of random-effects for pooled SMDs of best corrected visual acuity (BCVA) after uncomplicated phacoemulsification in both diabetic patients without diabetic retinopathy and in diabetic patients with non-proliferative diabetic retinopathy.

The pooled estimates of mean changes in BCVA (logMAR) after uncomplicated phacoemulsification in both diabetic patients without DR and diabetic patients with mild to moderate NPDR, along with SMD and 95%CI are showed in Fig 4. A significant improvement in BCVA was observed in patients without DR at postoperative 1 month (SMD,1.149; 95%CI, 0.251 to 2.047, P = 0.012) and 6 months (SMD, 1.295;95%CI, 0.494 to 2.096; P = 0.002). BCVA of diabetic patients with mild to moderate NPDR was significantly improved at postoperative 1 month (SMD, 1.349; 95%CI, 0.264 to 2.434; P = 0.015)and 6 months (SMD, 2.146; 95%CI, 0.172 to 4.120; P = 0.033).

### Sensitivity analysis and publication bias

**Fig 5:**
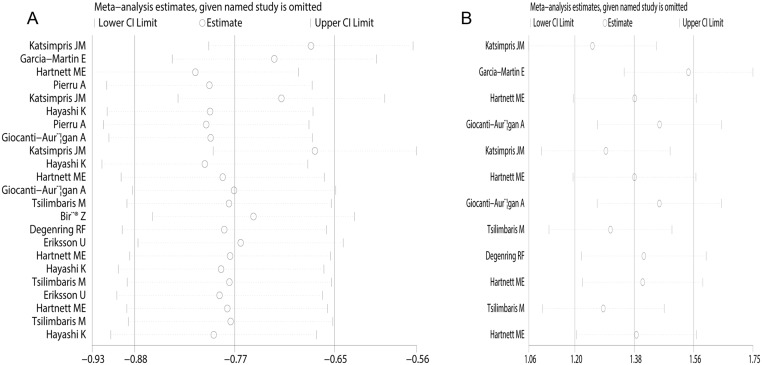
Meta- sensitivity analyses on central macular thickness (A) and best corrected visual acuity (B) in both diabetic patients without diabetic retinopathy and diabetic patients with non-proliferative diabetic retinopathy.

Sensitivity analysis was performed by consecutively removing each study from the statistic. We found that each individual study could not alter the pooled estimates of CMT values (SMD, -0.77; 95%CI, -0.88 to -0.65; Fig 5A) or BCVA (SMD, 1.38; 95%CI, 1.20 to 1.56; Fig 5B), which indicates that the results of this meta-analysis are robust. The Egger test showed no evidence of publication bias for CMT values (P = 0.570, t = -0.58) or BCVA (P = 0.952, t = 0.06).

## Discussion

This study provides robust evidence on the effect of uncomplicated phacoemulsification on CMT values in both diabetic patients without DR and diabetic patients with NPDR. Overall, there was a statistically significant increase in CMT values in diabetic patients with mild to moderate NPDR compared with diabetic patients without DR at postoperative 1month. Such an increase was still higher in diabetic patients with mild to moderate NPDR at postoperative 3 and 6 months. BCVA was significantly improved at postoperative 1 month and 6 months in both diabetic patients without DR and diabetic patients with mild to moderate NPDR.

Postoperative subclinical central macular thickening can often be seen without visual impairment, and it is detectable by angiographic examination [24]. Surgery itself can cause inflammatory response by releasing prostaglandins, which plays an important role in the occurrence of macular thickening [25]. CMT values in diabetic patients with NPDR showed a statistically significant increase at postoperative 1monthcompared with diabetic patients without DR. The results indicate that uncomplicated phacoemulsification has some effect upon the underlying pathophysiology of retinopathy.

Phacoemulsification is the most widely surgical technique that uses ultrasonic energy to remove the lens darkened by cloudy imperfections, and the clouded lens is then replaced with an intraocular lens [26]. Many studies have shown that phacoemulsification and intraocular lens implantation provide satisfactory visual outcomes through a smaller incision [26,27]. This makes cataract surgery safer, accelerates the recovery of vision, and decreasespostoperative complications [27].

We found that uncomplicated phacoemulsification significantly improved BCVAat postoperative 1 month and 6 months in both diabetic patients without DR and diabetic patients with mild to moderate NPDR. There was a significant increase in CMT values in diabetic patients with NPDR and a nearly significant increase (P = 0.054) in diabetic patients without DR at postoperative 1 month. The changes in CMT values may be due to the inflammatory response to uncomplicated phacoemulsification at postoperative 1 month and may also be associated with the pathologic features of Irvine-Gass syndrome caused by cytokines and growth factor (e.g., prostaglandins and VEGF) released from the blood—ocular barrier after cataract surgery.

Our study evaluated the changes in CMT values at 1, 3 and 6 months after uncomplicated phacoemulsification in both diabetic patients without DR and diabetic patients with mild to moderate NPDR. Diabetic patients with NPDR showed a significant increase in CMT values at postoperative 1,3 and 6 months. However, visual outcomes were not compromised in diabetic patients with mild to moderate NPDR at postoperative 1 month and 6 months, indicating that the changes in CMT values remained subclinical in diabetic patients with NPDR. No statistically significant increase in CMT values was observed at postoperative 3 and 6 months in diabetic patients without DR. The results showed that uncomplicated phacoemulsification surgery had little effect upon the underlying pathophysiology of retinopathy in diabetic patients without DR, and diabetic patients with mild to moderate NPDR had a higher incidence of subclinical macular thickening after uncomplicated phacoemulsification than diabetic patients without DR.

Diabetic patients may be susceptible to develop postoperative subclinical retinal swelling or clinical macular edema after cataract surgery[28]. The effect of uncomplicated phacoemulsification surgery on the progression of DR remains an issue. Some risk factors, such as young age, insulin therapy or poor control of blood glucose levels, may influence the postoperative progression of DR. However, Kato *et al* [29–31] demonstrated that these factors do not affect the progression of retinopathy. The pathogenesis of postoperative CME is involved in the intraocular inflammation occurring secondary to the release of prostaglandins in patients with and without diabetes [32].

VEGF plays an important role in the pathogenesis of diabetic microangiopathy, due to its ability to increase vascular permeability [33]. In addition, the level of VEGF in vitreous humour is significantly higher in patients with proliferative DR [34]. Thus, intravitreal ranibizumab application is a promising treatment [5,35]. It is important to distinguish diabetic macular edema from pseudophakic CME after cataract surgery in diabetic patients. This is especially true in the early postoperative period where it has been shown that pseudophakic CME is prone to regress when caused by Irvine-Gass syndrome but progress when caused by diabetes [36].

Surgically induced inflammatory responses caused early macular changes that lessened as the inflammation subsided almost 3 months after uncomplicated phacoemulsification [37–39]. We found that CMT values showed a significant difference after uncomplicated phacoemulsification at 1, 3 and 6 months in diabetic patients with NPDR. These findings indicate that diabetes has some influence on the changes of CMT values after uncomplicated phacoemulsification in diabetic patients with NPDR. Uncomplicated phacoemulsification with intraocular lens implantation affected the blood-aqueous barrier more severely in diabetic patients with mild to moderate NPDR than diabetic patients without DR. Several investigators have reported that CMT values after cataract surgery are increased as the surgical trauma induces a rapid increase in CMT values in eyes with a long history of maculopathy and preexisting edema[8,40].

Before this study, there was little robust evidence regarding the effect of uncomplicated phacoemulsification on the changes of CMT values in both diabetic patients without DR and diabetic patients with mild to moderate NPDR. The results of our meta-analysis show that CMT values are significantly higher in diabetic patients with mild to moderate NPDR after uncomplicated phacoemulsification compared with diabetic patients without DR. Currently, the two most widely used diagnostic imaging methods are “time-domain OCT (TD-OCT)” and “spectral domain OCT (SD-OCT)”. These two measurements can vary by as much as57 microns due to different measuring principles [41]. In our study, we compared the CMT values before and after cataract surgery in both diabetic patients without diabetic retinopathy and diabetic patients with mild to moderate NPDR, so the difference in CMT values before and after cataract surgery can be comparable through a combination of TD-OCT and SD-OCT techniques. These results suggest that uncomplicated phacoemulsification could influence the changes of CMT values in diabetic patients with different severities of retinopathy.

Limiting this study was the small number of trials involving diabetic patients without DR (n = 6) and diabetic patients with mild to moderate NPDR (n = 6). Furthermore, we did not include studies with patients who underwent complicated cataract surgery or those with pre-existing proliferative retinopathy. A 1-, 3- and 6-months follow-up study was conducted, however, longer follow-up may be necessary in order to assess the changes in CMT values.

In conclusion, this study shows that uncomplicated phacoemulsification with intraocular lens implantation causes a significant increase in subclinical thickening in the region of the central macula in diabetic patients with mild to moderate NPDR at postoperative 1, 3 and 6 months compared with diabetic patients without DR. BCVA was found to be better at postoperative 1 month and 6 monthsin both diabetic patients without DR and diabetic patients with mild to moderate NPDR. Well-designed studies with larger sample sizes and longer follow-up periods are warranted for further research and development.

## Supporting Information

S1 PRISMA Checklist PRISMA Checklist. (DOC)
